# The impact on clinical outcomes and healthcare resources from discontinuing colonoscopy surveillance subsequent to low-risk adenoma removal: A simulation study using the OncoSim-Colorectal model

**DOI:** 10.1177/09691413231202877

**Published:** 2023-09-20

**Authors:** Kieran JD Steer, Zhuolu Sun, Daniel C Sadowski, Jean H E Yong, Andrew Coldman, Nicole Nemecek, Huiming Yang

**Affiliations:** 1 Department of Community Health Sciences, 2129 University of Calgary, Calgary, AB, Canada; 2 Provincial Population and Public Health, 3146 Alberta Health Services, Calgary, AB, Canada; 3 137691 Canadian Partnership Against Cancer, Toronto, ON, Canada; 4 Division of Gastroenterology, 12357 University of Alberta Faculty of Medicine and Dentistry, Edmonton, AB, Canada; 5 Cancer Control Research, 8162 British Columbia Cancer Research Centre, Vancouver, BC, Canada

**Keywords:** Colorectal cancer, screening, simulation, surveillance

## Abstract

**Objective:**

To estimate the impact on clinical outcomes and healthcare resource use from recommending that patients with 1–2 low-risk adenomas (LRAs) return to routine fecal immunochemical test (FIT) screening instead of surveillance colonoscopy, from a Canadian provincial healthcare system perspective.

**Methods:**

The OncoSim-Colorectal microsimulation model simulated average-risk individuals eligible for FIT-based colorectal cancer (CRC) screening in Alberta, Canada. We simulated two surveillance strategies that applied to individuals with 1–2 LRAs (<10 mm) removed as part of the average risk CRC screening program: (a) Surveillance colonoscopy (status quo) and (b) return to FIT screening (new strategy); both at 5 years after polypectomy. A 75 ng/mL FIT positivity threshold was used in the base case. The simulations projected average annual CRC outcomes and healthcare resource use from 2023 to 2042. We conducted alternative scenarios and sensitivity analyses on key variables.

**Results:**

Returning to FIT screening (versus surveillance colonoscopy) after polypectomy was projected to have minimal impact on long-term CRC incidence and deaths (not statistically significant). There was a projected decrease of one (4%) major bleeding event and seven (5%) perforation events per year. There was a projected increase of 4800 (1.5%) FIT screens, decrease of 3900 (5.1%) colonoscopies, and a decrease of $3.4 million (1.2%) in total healthcare costs per year, on average. The annual colonoscopies averted and healthcare cost savings increased over time. Results were similar in the alternative scenarios and sensitivity analyses.

**Conclusions:**

Returning to FIT screening would have similar clinical outcomes as surveillance colonoscopy but could reduce colonoscopy demand and healthcare costs.

## Introduction

Cancer screening programs aim to improve population health by systematically testing at-risk asymptomatic individuals to identify and treat cases in early or pre-clinical stages.^1^ Colorectal cancer (CRC) screening with fecal immunochemical test (FIT) effectively detects cancers at early stages and identifies pre-malignant lesions to decrease CRC mortality and incidence.^2–4^ Substantial healthcare resources are required to screen populations and manage the high volume of identified lesions, so optimizing resource use for screening programs is important.^5^ Currently, screening with FIT is offered to average-risk Alberta residents aged 50–74 with a screening interval of 1–2 years and a threshold of 75 ng/mL to indicate further follow-up.^6^ People with a significant family history of CRC may be screened directly with colonoscopy, due to an elevated CRC risk.^6^

Adenoma management is an important aspect of CRC screening pathways.^7^ Different types of adenomas have different risks of progressing to cancer. Individuals with resected large adenomas (≥10 mm) have a significantly increased CRC risk,^8–10^ while individuals with resected small adenomas (<10 mm) do not.^11^ Small adenomas (<10 mm) are typically referred to as low-risk adenomas (LRAs). A recent meta-analysis of CRC outcomes in patients with LRAs found that people with 1–2 LRAs resected on a screening colonoscopy had a decreased CRC incidence and risk of CRC-related death compared to the age- and sex-matched general population.^12^ As a result of this emerging evidence, many jurisdictions now recommend returning to FIT screening instead of providing a surveillance colonoscopy for patients with 1–2 LRAs.^13–18^ The impact of this change in post-polypectomy surveillance strategy on long-term healthcare system costs and equitable distribution of healthcare resources in the Canadian health care system is currently unknown.

The study objective was to estimate the changes in clinical outcomes and healthcare resource use from recommending that patients with 1–2 LRAs return to FIT-based screening in 5 years (new strategy) instead of receiving a surveillance colonoscopy in 5 years (status quo), from a Canadian provincial healthcare system perspective.

## Methods

All reporting followed STrengthening the Reporting of OBservational studies in Epidemiology (STROBE) and Simulation-Based Research Extensions for the STROBE Statement.^19^

### OncoSim-Colorectal model

The OncoSim-Colorectal version 3.4.5.8 was used to investigate this research question. OncoSim is a mathematical microsimulation cancer modeling tool developed by the Canadian Partnership Against Cancer with support from Statistics Canada. OncoSim creates “histories” of one individual at a time to mimic the demographics of the Canadian population using demography data (birth, migration and immigration by sex, province/ territory, and year) from Statistics Canada. The model is calibrated to match the CRC incidence and deaths in the Canadian Cancer Registry. The development, application, and validation of the OncoSim models have been described in detail in an earlier publication.^20^ The structure of OncoSim-Colorectal is similar to other models from other countries simulating the CRC natural history.^21^ An earlier publication demonstrated that OncoSim-Colorectal reproduces the effectiveness of CRC screening that was observed in several randomized clinical trials.^22^

OncoSim-Colorectal models the natural history of colorectal polyps and cancers (Supplemental Figure 1),^20,22^ and there are some key assumptions in the model regarding LRAs. Adenomas are assumed to spontaneously develop, and can either grow in size, revert back to normal mucosa, or progress to CRC. Adenomas are categorized into three size groups: 0–5, 6–9, or ≥10 mm. The model assumes that all adenomas (including adenomas <10 mm) have elevated risk of transition to CRC, in comparison to normal mucosa. All adenomas and cancers are potentially detectable by FIT, and the probability of a positive FIT is based on a patient's most advanced lesion at the time of the FIT.

### Study design

The model simulated the entire population in the province of Alberta, Canada aged 50–74 years, which is the age range that is eligible for FIT-based CRC screening in Alberta. The number of simulated participants eligible for CRC screening by FIT was 1,190,189 people in 2023. Participants were followed until their death. We assumed a provincial FIT-based screening program had been in place since 2008.

This study compared two surveillance strategies for patients with LRA, defined as tubular adenomas <10 mm with no dysplasia on histology, after polypectomy (Table 1):

1. colonoscopy in 5 years (status quo), and
2. annual/biennial FIT screening commencing in 5 years (new strategy).

**Table 1.** Descriptions of the status quo and new strategy.

Surveillance strategy	Description
Colonoscopy surveillance (status quo)	Colonoscopy in 5 years for patients with 1–2 LRAs on colonoscopy
Return to FIT screening (new strategy)	Return to average-risk screening with FIT in 5 years for patients with 1–2 LRAs on colonoscopy

LRA was defined as an adenoma <10 mm with no dysplasia on histology. FIT: fecal immunochemical testing; LRA: low-risk adenoma.

Patients with any villous adenoma, any tubular adenoma ≥10 mm, or >2 LRAs received a surveillance colonoscopy in 3 years and then 5 years after. Patients with a normal colonoscopy returned to average risk screening with FIT 10 years after the normal colonoscopy. We assumed that the change in follow-up strategy in 2023 only affected patients who received their initial diagnostic colonoscopy in the year 2023 or later.

Outcomes were followed over a 20-year time horizon starting in 2023, meaning that only costs and outcomes incurred during the years 2023–2042 were included. A 20-year time horizon was used to inform guidelines and healthcare decision-makers on the changes in colonoscopy demand, healthcare costs, and patient outcomes in the short to medium term. The analysis was conducted from the perspective of a public healthcare payer and included healthcare costs for the public payer. Productivity losses associated with illness and caregiving and out-of-pocket costs for patients and families were not included.

### Model inputs

A summary of model inputs is provided in Table 2. Participants had an overall screening participation rate of 42% in 2019, which was calibrated to match internal Alberta Health Services data from 2019 (unpublished). Screening frequency was 50% annual and 50% biennial to represent the Alberta recommendation of FIT screening every 1–2 years. We assumed that FIT was used as the primary screening method, and colonoscopies were provided after a positive FIT or for surveillance after adenomas were found on colonoscopy. The incidence of LRA on colonoscopy was set to 26/100 colonoscopies, which is the observed incidence from colonoscopy reports of FIT-positive patients in Alberta.

**Table 2.** Model inputs and costs used in the base case analysis.

Model inputs		Value	References
Screening frequency		50% annual 50% biennial	Based on Alberta recommendation of FIT every 1–2 years
Compliance to FIT or colonoscopy after polypectomy		100%	Conservative assumption due to lack of evidence around the outcomes among people with partial screening participation
Overall screening participation rate		42%	Internal Alberta Health Services data from 2019 (unpublished)
FIT positivity threshold		75 ng/mL	Based on current Alberta threshold
LRA incidence (per 100 colonoscopies)		26	Observed incidence from colonoscopy among FIT-positive patients in Alberta (unpublished)
Colonoscopy complication rate (per 1000 colonoscopies)	Major bleeding	0.3	Major bleeding and perforation rates from Habr-Gama et al. 1989 ^21^ Mortality rate from Vermeer et al. 2017 ^23^
	Perforation	1.7	
	Mortality	0.0	
Cost category		Cost (2021 CAD)	Cost components
Physician recruitment to CRC screening		$0	Assumption that it's part of general preventive care
FIT screen		$26.13	Kit cost and processing SMB E248A (general practitioner ordering FIT)
Physician extra visits to discuss FIT results (cost per screen)		$38.03	SMB 03.03A (general practitioner)
Consultation fee for positive test		$146.03	50% from SMB 03.08A (gastroenterology) 50% from SMB 03.07A (gastroenterology)
Diagnostic colonoscopy		$866.43	SMB 01.22A (gastroenterology) SMB 01.22A (anesthesia) Facility costs
Operative colonoscopy		$1036.43	SMB 01.22A (gastroenterology) SMB 01.22A (anesthesia) SMB 57.21A (assuming an average of 2 polypectomies per operative colonoscopy) Facility costs
Bleeding complication		$3864.74	Calculated by Heitman *et al.* 2010 ^24^
Perforation complication		$37,779.83	Calculated by Heitman *et al.* 2010 ^24^

CAD: Canadian dollars; CRC: colorectal cancer; FIT: fecal immunochemical test; LRA: low-risk adenoma; SMB: Alberta Health Schedule of Medical Benefits.

The base case used a FIT threshold of 75 ng/mL to signify a positive test, which is the threshold currently used in Alberta. A threshold of 100 ng/mL was used in a sensitivity analysis (Supplemental Table 1). For adenomas ≥10 mm and CRC, the FIT sensitivity rates of two thresholds (75 ng/mL and 100 ng/mL) were derived from a systematic review of FIT sensitivity.^25^ The sensitivities for smaller polyps are not well reported in the published literature. The sensitivities of the 75 ng/mL threshold for detecting smaller adenomas (0–5 and 6–9 mm) were calibrated to match Alberta data from 2019. For the 100 ng/mL threshold, we applied the relative sensitivities from the study by Coldman et al.^22^ to the calibrated values from 75 ng/mL to calculate the FIT sensitivities for adenomas smaller than 10 mm.

The incidence rate of colonoscopy complications was as follows: Major bleeding 0.3/1000 colonoscopies, perforation 1.7/1000 colonoscopies. We assumed there was no mortality from colonoscopies, based on results from a recent systematic review on colonoscopy screening-related mortality.^23^

Facility fees were estimated using internal Alberta Health Services costing data (unpublished), and physician fees were estimated through the Alberta Health Schedule of Medical Benefits effective February 1, 2022 (Table 2).^26^ The appropriate billing codes were verified via consultation with a gastroenterologist in Alberta and screening program administrative staff.

### Outcome variables

Clinical outcomes included CRC cases, CRC deaths, colonoscopy major bleeding events, and colonoscopy perforation events.

Healthcare resource use included healthcare costs, number of FIT screens, and number of colonoscopies. Healthcare costs were divided into three groups: Screening, clinical diagnosis, and cancer management. There were five categories of colonoscopies that contributed to different costs. Cost of screening included screening colonoscopies without FIT (e.g. for patients with a significant family history of CRC) and colonoscopies after a positive FIT. Clinical diagnosis costs included diagnostic colonoscopies for symptomatic patients, surveillance colonoscopies after adenoma detection, and surveillance colonoscopies after cancer detection.

### Alternative scenarios and sensitivity analyses

The following alternative scenarios were conducted: 100 ng/mL FIT positivity threshold, 100% biennial FIT screening frequency, and 100% annual FIT screening frequency. The following one-way sensitivity analyses were also conducted: alternative estimates of colonoscopy costs and alternative colonoscopy complication incidence rates (Supplemental Table 2).

### Statistical analysis

Incremental outcomes were calculated using “colonoscopy surveillance” as the status quo and “return to FIT screening” as the new strategy. To characterize the simulation uncertainty, we constructed confidence intervals (95%) for average annual incremental CRC incidence and death using the Monte Carlo errors. Each scenario was run 12 times, and we reported the mean and confidence intervals (calculated via a t-test) from the 12 sets of simulations, as done in other studies using OncoSim.^27^ Incremental major bleeds and perforation from colonoscopy were calculated by multiplying the incremental number of colonoscopies by the incidence rate per colonoscopy of major bleeds and perforation. Confidence intervals and statistical significance were not calculated for incremental healthcare resource use because the direction of the outcome (i.e. zero, positive, or negative) was predictable in the simulations.

All costs are expressed in 2021 Canadian dollars. This study aimed to quantify the projected changes in patient clinical outcomes and healthcare resource use, so no discounting was applied to costs or outcomes.

## Results

There was no statistically significant difference in average annual CRC cases or deaths between the return to FIT screening and colonoscopy surveillance strategies (Table 3). The return to the FIT screening strategy, compared to the colonoscopy surveillance strategy, had a decrease in the average annual incidence of major bleed and perforation events.

**Table 3.** Projected patient outcomes for each LRA surveillance strategy.

	Colonoscopy surveillance (status quo)	Return to FIT screening (new strategy)	Difference between strategies (95%CI)
CRC cases	2554	2571	16 (−2, 34)
CRC deaths	804	808	4 (−2, 11)
Major bleed from colonoscopy	23	22	−1 (4%)^a^
Perforation from colonoscopy	131	124	−7 (5%)^a^

Differences are expressed as “colonoscopy surveillance” as the status quo and “return to FIT screening” as the new strategy. Results are for the entire CRC screening-eligible population in Alberta, expressed as average annual values over 20 years (2023–2042). CRC: colorectal cancer, FIT: fecal immunochemical test; LRA: low-risk adenomas.

^a^ Reported as the difference and percentage change between strategies, compared to the status quo.

The return to FIT screening strategy had an increased annual number of FIT screens and a decreased total number of colonoscopies (Table 4). The return to FIT screening strategy had increases in the number of colonoscopies after a positive FIT, diagnostic colonoscopies for symptomatic patients, and surveillance colonoscopies after cancer detection. The return to FIT screening strategy had a decreased number of surveillance colonoscopies after adenoma.

**Table 4.** Projected number of FIT screens and colonoscopies for each LRA surveillance strategy.

		Colonoscopy surveillance (status quo)	Return to FIT screening (new strategy)	Difference between strategies (% change)
Screening	FIT screens	323,809	328,597	4788 (1.5%)
	Screening colonoscopies without FIT	16,080	16,080	0 (0.0%)
	Colonoscopies after a positive FIT	25,774	26,295	521 (2.0%)
Clinical diagnosis	Colonoscopies for symptomatic patients	1863	1882	19 (1.0%)
	Surveillance colonoscopies after adenoma detection	23,286	18,837	−4449 (−19.1%)
Cancer management	Surveillance colonoscopies after cancer detection	9898	9915	17 (0.2%)
Total number of colonoscopies		76,901	73,009	−3892 (−5.1%)

Differences are expressed as “colonoscopy surveillance” as the status quo and “return to FIT screening” as the new strategy. Results are for the entire CRC screening-eligible population in Alberta, expressed as average annual value over 20 simulated years (2023–2042). CRC: colorectal cancer, FIT: fecal immunochemical test; LRA: low-risk adenomas.

The return to FIT screening strategy had increased cost of screening, decreased the cost of clinical diagnosis (which included surveillance colonoscopies), and increased cost of cancer management in both FIT threshold scenarios (Table 5). The return to FIT screening strategy decreased average annual total healthcare cost by $3.4 million (1.2% savings).

**Table 5.** Projected impact on healthcare costs for each LRA surveillance strategy.

	Colonoscopy surveillance (status quo)	Return to FIT screening (new strategy)	Difference between strategies (% change)
Cost of screening	$86,743,719	$88,131,887	$1,388,168 (1.6%)
Cost of clinical diagnosis	$50,189,444	$44,463,946	−$5,725,497 (−11.4%)
Cost of cancer management	$148,726,702	$149,628,663	$901,960 (0.6%)
Total cost (screening and diagnostics)	$136,933,163	$132,595,834	−$4,337,329 (−3.2%)
Total cost (all categories)	$285,659,865	$282,224,496	−$3,435,369 (−1.2%)

Differences are expressed as “colonoscopy surveillance” as the status quo and “return to FIT screening” as the new strategy. Results are for the entire CRC screening-eligible population in Alberta, expressed as average annual value over 20 simulated years (2023–2042). CRC: colorectal cancer, FIT: fecal immunochemical test; LRA: low-risk adenomas.

The annual colonoscopies averted and healthcare cost savings began to occur 5 years after switching to the return to FIT screening strategy and gradually increased afterward (Supplemental Figure 2). The incremental number of CRC cases per 1000 surveillance colonoscopies averted was 3.6 [95%CI −0.4, 7.6].

Results from the 100 ng/mL FIT threshold, 100% biennial screening, and 100% annual screening scenarios were similar to the base case (Supplemental Tables 3–5). The sensitivity analysis with lower colonoscopy costs resulted in smaller annual cost savings of $2.1 million (0.8% savings). The analysis with higher colonoscopy costs resulted in larger annual cost savings of $7.1 million (2.0% savings) (Supplemental Table 5). The sensitivity analysis with alternative colonoscopy complication rates had similar incremental major bleeding events to the base case but resulted in no incremental perforations (Supplemental Table 6).

## Discussion

We used the OncoSim-Colorectal microsimulation model to estimate the incremental clinical outcomes and healthcare resource use from return to FIT screening versus surveillance colonoscopy strategies among patients with 1–2 LRAs. There was no statistically significant difference in the average annual number of CRC cases or deaths, but there was a small decrease in annual colonoscopy complications. The return to FIT screening strategy had an average annual increase of 4800 (1.5%) FIT screens, decrease of 3900 (5.1%) colonoscopies, and a decrease of $3.4 million (1.2% savings) in total healthcare costs. The annual number of colonoscopies averted and healthcare cost savings increased over time. Results were similar in alternative scenarios using 100 ng/mL FIT threshold or exclusively biennial or annual FIT screening frequency and in sensitivity analyses with alternative colonoscopy costs and colonoscopy complication rates.

Our analysis found that the return to FIT screening strategy could lead to a very small but non-statistically significant increase in cancer incidence and deaths. Other modeling analyses also suggested a small increase but did not report the statistical significance of their results.^28,29^ A retrospective cohort study by Cross et al.^30^ found no significant difference in CRC incidence between patients with 1–2 LRAs who returned to FIT screening versus the age- and sex-matched general population (using United Kingdom CRC incidence rates). Providing one surveillance colonoscopy within 8.5 years for patients with 1–2 LRAs led to decreased CRC incidence, compared to the general population. However, the 10-year CRC cumulative incidence remained very low among people with 1–2 LRAs whether they received no surveillance (1.7% (95%CI 1.4–2.1)) or received one surveillance colonoscopy (1.5% (95%CI 1.0–2.3). Therefore, surveillance colonoscopy likely provides very small to no absolute difference in CRC incidence and is unlikely to affect CRC health outcomes at the population level, as seen in our study. Given the substantial clinical importance of any increases in CRC incidence in the population, jurisdictions should closely monitor CRC incidence if they change guidelines to not recommend surveillance colonoscopies after LRA.

Our study also found that the return to FIT screening strategy had a small decrease in average annual incidence of major bleeding and either a small decrease or no change in perforations, which is consistent with other studies.^31^ Our results also show that a 100 ng/mL FIT threshold, 100% biennial FIT screening, or 100% annual FIT screening strategies will likely have similar impacts on clinical outcomes or healthcare resource use from providing FIT screening instead of colonoscopy surveillance among patients with 1–2 LRAs.

Our study found that the return to FIT screening strategy had decreases in total healthcare costs and total colonoscopies, which were both primarily driven by a nearly 20% reduction in number of surveillance colonoscopies after adenoma detection. Other modeling analyses also found that using FIT screening instead of colonoscopy surveillance for individuals who had 1–2 LRAs and polypectomy could be cost-saving. A recent report by the Cancer Council of New South Wales in Australia^28^ and two recent cost-effectiveness analyses^29,30^ used microsimulation or Markov modeling approaches and similarly found decreases in average annual colonoscopy use and total healthcare cost by using FIT screening instead of colonoscopy surveillance among people with 1–2 LRAs. The Australian report found percentage decreases in average annual colonoscopy use (8.9%) and total healthcare costs (1.2%) that were similar to the decreases in our study.

The return to FIT screening strategy led to 3900 (5.1%) fewer annual colonoscopies, which has other important benefits that could not be measured in the OncoSim model. Fewer annual colonoscopies may reduce colonoscopy wait times, thereby reducing patient anxiety^32^ and preventing delays in CRC diagnosis.^33,34^ Colonoscopy wait times throughout Canada currently exceed the medically recommended time periods for patients with positive FIT^35–37^ and for patients with CRC symptoms,^34,38^ which has been exacerbated by interruptions in medical services due to the coronavirus disease 2019 pandemic.^39^ Fewer annual colonoscopies may also reduce patient's time lost from normal activities^40^ and reduce colonoscopy-related morbidity.^23^

### Study limitations

All data were generated through simulations using mathematical risk equations and are not new primary data. The results may have limited generalizability in other jurisdictions because all data inputs were selected to maximize generalizability to the Alberta context. The OncoSim model was not able to incorporate some variables, such as screening compliance and appointment no-shows. Cost inputs are also subject to real-world variations that are challenging to estimate, such as variations in clinical practices and changes in healthcare system efficiency over time. However, our sensitivity analyses showed that there were substantial annual cost savings of $2.1 million even with the lowest estimates of colonoscopy costs. The study assumed that return to FIT screening only applied to people who received a diagnostic colonoscopy in 2023 and onwards, so colonoscopies averted and cost savings were delayed for approximately 5 years. In real life, the policy might be applied to people who are due for surveillance in 2023; therefore, the impact on colonoscopies would be immediate (i.e. starting in 2023).

## Conclusion

Simulations of return to FIT screening instead of surveillance colonoscopy for patients with 1–2 LRAs led to no statistically significant differences in average annual CRC incidence or death and a small decrease in colonoscopy complications. There was an average annual increase in FIT screens (4800 (1.5%)), decrease in colonoscopies (3900 (−5.1%)), and a decrease in total healthcare costs ($3.4 million (−1.2%)). The results show there are similar clinical outcomes and overall reductions in healthcare system resource use from returning to FIT screening in patients with 1–2 LRAs. Monitoring actual clinical outcomes and resource impacts will be important if this change in follow-up strategy is implemented.

## Supplemental Material

sj-pdf-1-msc-10.1177_09691413231202877 - Supplemental material for The impact on clinical outcomes and healthcare resources from discontinuing colonoscopy surveillance subsequent to low-risk adenoma removal: A simulation study using the OncoSim-Colorectal model Supplemental material, sj-pdf-1-msc-10.1177_09691413231202877 for The impact on clinical outcomes and healthcare resources from discontinuing colonoscopy surveillance subsequent to low-risk adenoma removal: A simulation study using the OncoSim-Colorectal model by Kieran JD Steer, Zhuolu Sun, Daniel C Sadowski and Jean H E Yong, Andrew Coldman, Nicole Nemecek, Huiming Yang in Journal of Medical Screening
