# Supplementary material for: The impact on clinical outcomes and healthcare resources from discontinuing colonoscopy surveillance subsequent to low-risk adenoma removal: A simulation study using the OncoSim-Colorectal model
Source: J Med Screen. 2023 Sep 20;31(2):78–84. doi: 10.1177/09691413231202877 (PMC11083724; doi:10.1177/09691413231202877)
Supplement: sj-pdf-1-msc-10.1177_09691413231202877 - Supplemental material for The impact on clinical outcomes and healthcare resources from discontinuing colonoscopy surveillance subsequent to low-risk adenoma removal: A simulation study using the OncoSim-Colorectal model [file sj-pdf-1-msc-10.1177_09691413231202877.pdf]

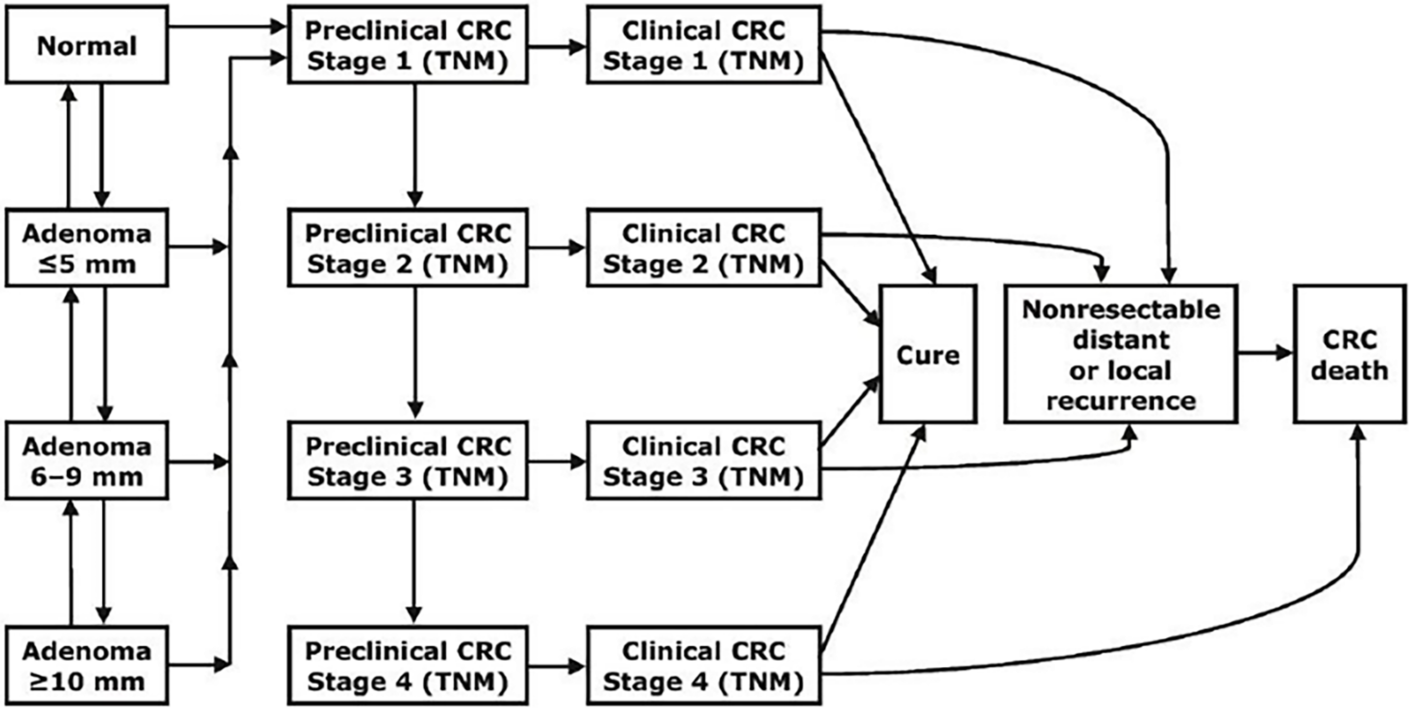

Supplemental Figure 1: Schematic diagram of the OncoSim-Colorectal model

Supplemental Table 1: True positive rate for FIT at 75 ng/mL and 100 ng/mL test positivity thresholds

	**75 ng/mL**	**100 ng/mL**
Polyp ≤ 5mm	0.15	0.11
Polyp between 6-9 mm	0.2	0.15
Polyp ≥ 10mm	0.3	0.26
CRC	0.81	0.77

CRC: colorectal cancer; FIT: fecal immunochemical test.

Supplemental Table 2: List of alternative scenarios and sensitivity analyses

**Alternative scenarios**	**Base case assumption**	**Scenario assumption**	**Data source**
Scenario 1: FIT positivity threshold	75 ng/mL	100 ng/mL starting in 2023	Alberta is considering changing FIT positivity threshold to 100 ng/mL
Scenario 2: biennial FIT screening frequency	50% annual<br>50% biennial	100% biennial starting in 2023	Alberta is considering changing recommendation to biennial FIT screening
Scenario 3: annual FIT screening frequency		100% annual starting in 2023	Annual FIT screening frequency is frequently used in other jurisdictions [1]
**Parameter for sensitivity analysis**	**Parameter in base case**	**Parameter in sensitivity analysis**	**Data source**
Scenario 4: Alternative colonoscopy costs	Diagnostic: $866.43<br>Operative: $1036.43	Lower costs:<br>Diagnostic: $605<br>Operative $847	Range derived from Heitman *et al.* [2]

		Higher costs:<br><br>Diagnostic: $1452<br><br>Operative: $2057	Costs inflated to 2021 CAD using the health and personal care portion of the Consumer Price Index [3]
Scenario 5: Alternative colonoscopy complication rates	Major bleeding: 0.3/1000 colonoscopies<br><br>Perforation: 1.7/1000 colonoscopies	Major bleeding: 0.8/1000 colonoscopies<br><br>Perforation: 0.07/1000 colonoscopies	Alternative colonoscopy complication rates from a meta-analysis by Vermeer *et al.* 2017 [4]

CAD: Canadian dollars; FIT: fecal immunochemical test.

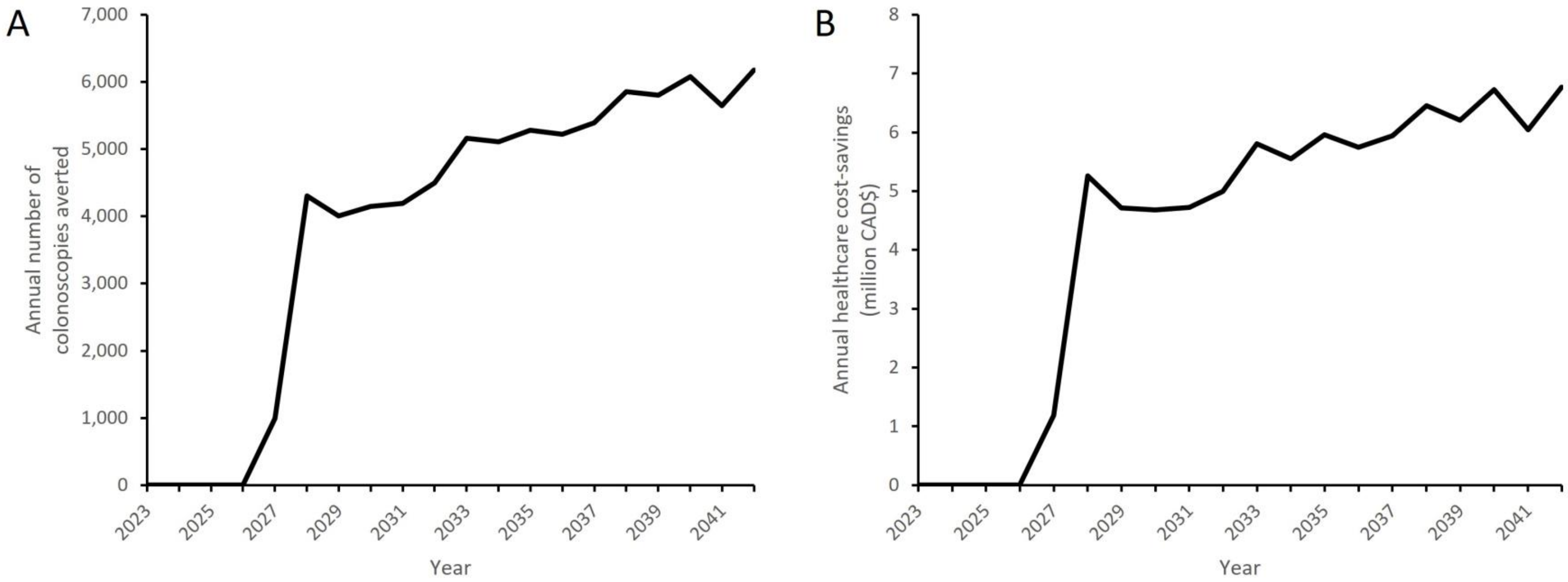

Supplemental Figure 2: Trend over 20 simulated years (2023-2042) of the A: annual number of colonoscopies averted, and B: annual healthcare cost savings (from screening and clinical diagnosis), from the return to FIT screening strategy (with “colonoscopy surveillance” as the status quo).

Supplemental Table 3: Projected patient outcomes for scenarios in one-way sensitivity analyses. Differences are expressed as “colonoscopy surveillance” as the status quo and “return to FIT screening” as the new strategy. Results are for the entire CRC screening-eligible population in Alberta, expressed as average annual values over 20 years (2023-2042).

	**Scenario 1: 100 ng/mL FIT threshold**			**Scenario 2: 100% biennial FIT**			**Scenario 3: 100% annual FIT**		
	**Colonoscopy surveillance (status quo)**	**Return to FIT screening (new strategy)**	**Difference between strategies (95%CI)**	**Colonoscopy surveillance (status quo)**	**Return to FIT screening (new strategy)**	**Difference between strategies (95%CI)**	**Colonoscopy surveillance (status quo)**	**Return to FIT screening (new strategy)**	**Difference between strategies (95%CI)**
CRC cases	2,642	2,656	14 (-2, 31)	2,651	2,665	14 (-2, 31)	2,396	2,417	21 (-1, 42)
CRC deaths	829	832	4 (-2, 9)	852	855	3 (-3, 8)	750	755	4 (-3, 12)
Major bleed from colonoscopy	22	21	-1 (-5%)*	21	20	-1 (-5%)*	26	25	-1 (-5%)
Perforation from colonoscopy	123	117	-6 (-5%)*	120	115	-5 (-5%)*	148	140	-8 (-5%)

*Reported as the difference and percentage change between strategies, compared to the status quo.
CRC: colorectal cancer, FIT: fecal immunochemical test.

Supplemental Table 4: Projected number of FIT screens and colonoscopies for scenarios in one-way sensitivity analyses. Differences are expressed as "colonoscopy surveillance" as the status quo and "return to FIT screening" as the new strategy. Results are for the entire CRC screening-eligible population in Alberta, expressed as average annual value over 20 simulated years (2023-2042).

		**Scenario 1: 100 ng/mL FIT threshold**			**Scenario 2: 100% biennial FIT**			**Scenario 3: 100% annual FIT**		
		**Colonoscopy surveillance (status quo)**	**Return to FIT screening (new strategy)**	**Difference between strategies (% change)**	**Colonoscopy surveillance (status quo)**	**Return to FIT screening (new strategy)**	**Difference between strategies (% change)**	**Colonoscopy surveillance (status quo)**	**Return to FIT screening (new strategy)**	**Difference between strategies (% change)**
Screening	FIT screens	329,214	332,992	3,778 (1.1%)	262,833	266,304	6,943 (2.6%)	437,120	446,303	16,902 (3.9%)
	Screening colonoscopies without FIT	16,080	16,080	0 (0.0%)	16,080	16,080	0 (0.0%)	16,080	16,080	0 (0.0%)
	Colonoscopies after a positive FIT	22,721	23,054	332 (1.5%)	21,272	21,650	378 (1.8%)	33,629	34,571	942 (2.8%)

Clinical diagnosis	Colonoscopies for symptomatic patients	1,949	1,964	15 (0.8%)	2,027	2,045	18 (0.9%)	1,679	1,701	22 (1.3%)
	Surveillance colonoscopies after adenoma detection	21,376	17,893	-3,483 (-16.3%)	21,466	17,750	-3,717 (-17.3%)	26,109	20,454	-5,655 (-21.7%)
Cancer management	Surveillance colonoscopies after cancer detection	10,012	10,025	13 (0.1%)	9,961	9,975	14 (0.1%)	9,701	9,727	26 (0.3%)
	Total number of colonoscopies	72,138	69,016	-3,122 (-4.3%)	70,806	67,500	-3,306 (-4.7%)	87,198	82,533	-4,664 (-5.3%)

CRC: colorectal cancer, FIT: fecal immunochemical test.

Supplemental Table 5: Projected difference in healthcare costs between return to FIT screening (new strategy) versus colonoscopy surveillance (status quo) for scenarios in the repeated one-way sensitivity analysis. Results are for the entire CRC screening-eligible population in Alberta, expressed as average annual values over 20 years (2023-2042).

	**Scenario 1: 100 ng/mL FIT threshold**	**Scenario 2: 100% biennial screening**	**Scenario 3: 100% annual screening**	**Scenario 4: Lower colonoscopy costs**	**Scenario 5: Higher colonoscopy costs**
Cost of screening	$955,679 (1.2%)	$1,007,060 (1.4%)	$2,516,360 (2.3%)	$1,187,564 (1.6%)	$2,040,245 (1.7%)
Cost of clinical diagnosis	-$4,493,917 (-9.4%)	-$4,787,340 (-9.9%)	-$7,281,585 (-13.6%)	-$4,258,216 (-11.3%)	-$10,000,316 (-11.3%)
Cost of cancer management	$768,193 (0.5%)	$753,149 (0.5%)	$1,082,233 (0.8%)	$901,960 (0.6%)	$901,960 (0.6%)
Total cost (screening and diagnostics)	-$3,538,238 (-2.7%)	-$3,780,280 (-3.1%)	-$4,765,225 (-2.9%)	-$3,070,651 (-2.8%)	-$7,960,071 (-3.8%)
Total cost (all categories)	-$2,770,044 (-1.0%)	-$3,027,131 (-1.1%)	-$3,682,992 (-1.2%)	-$2,168,691 (-0.8%)	-$7,058,111 (-2.0%)

CRC: colorectal cancer, FIT: fecal immunochemical test.

Supplemental Table 6: Projected colonoscopy complications using alternative colonoscopy complication rates. Differences are expressed as “colonoscopy surveillance” as the status quo and “return to FIT screening” as the new strategy. Results are for the entire CRC screening-eligible population in Alberta, expressed as average annual values over 20 years (2023-2042).

	**Colonoscopy surveillance (status quo)**	**Return to FIT screening (new strategy)**	**Difference between strategies (% change from status quo)**
Major bleed from colonoscopy	62	58	-3 (-5%)
Perforation from colonoscopy	5	5	0 (0%)

FIT: fecal immunochemical test.

References

1. Ebell MH, Thai TN and Royalty KJ. Cancer screening recommendations: an international comparison of high income countries. *Public Health Rev* 2018; 39: 7. 20180302. DOI: 10.1186/s40985-018-0080-0.

2. Heitman SJ, Hilsden RJ, Au F, et al. Colorectal cancer screening for average-risk North Americans: an economic evaluation. *PLoS Med* 2010; 7: e1000370. 20101123. DOI: 10.1371/journal.pmed.1000370.

3. Statistics Canada. *Consumer price index portal*. 2021.

4. Vermeer NC, Snijders HS, Holman FA, et al. Colorectal cancer screening: Systematic review of screen-related morbidity and mortality. *Cancer Treat Rev* 2017; 54: 87-98. 20170216. DOI: 10.1016/j.ctrv.2017.02.002.
